# Objective Assessment of the Core Laparoscopic Skills Course

**DOI:** 10.1155/2012/379625

**Published:** 2012-05-08

**Authors:** Sami Mansour, Nizar Din, Kumaran Ratnasingham, Shashidhar Irukulla, George Vasilikostas, Marcus Reddy, Andrew Wan

**Affiliations:** Upper Gastrointestinal Surgery Department, St. George's Healthcare NHS Trust, London SW17 0QT, UK

## Abstract

*Objective.* The demand for laparoscopic surgery has led to the core laparoscopic skills course (CLSC) becoming mandatory for trainees in UK. Virtual reality simulation (VR) has a great potential as a training and assessment tool of laparoscopic skills. The aim of this study was to determine the role of the CLSC in developing laparoscopic skills using the VR. *Design.* Prospective study. Doctors were given teaching to explain how to perform PEG transfer and clipping skills using the VR. They carried out these skills before and after the course. During the course they were trained using the Box Trainer (BT). Certain parameters assessed. *Setting.* Between 2008 and 2010, doctors attending the CLSC at St Georges Hospital. *Participants.* All doctors with minimal laparoscopic experience attending the CLSC. *Results*. Forty eight doctors were included. The time taken for the PEG skill improved by 52%, total left hand and right hand length by 41% and 48%. The total time in the clipping skill improved by 57%. Improvement in clips applied in the marked area was 38% and 45% in maximum vessel stretch. *Conclusions.* This study demonstrated that CLSC improved some aspects of the laparoscopic surgical skills. It addresses Practice-based Learning and patient care.

## 1. Introduction and Objective

Laparoscopic surgery is technically demanding and requires psychomotor skills different from those needed in open surgery. Training in laparoscopic surgery is done in the operating theatre but in the future we have to expect increasing focus on ethics and patient safety. This might demand better and more intensive training in a safer environment prior to training in the operating theatre. Recently, the acquisition of such skills has been via didactic lectures and simulator training [1], which is provided in the Core Laparoscopic Skills Course (CLSC).

A wide variety of laparoscopic simulators are now available, and they can be broadly classified into videoscopic and computer-driven laparoscopic simulation platforms, which are further divided into virtual reality (VR) and computer-enhanced videoscopic trainers. These trainers primarily differ in their user interface and ability to provide reliable performance measurements. Videoscopic trainer allows manipulation of actual physical objects and requires manual data collection. In contrast, VR trainer utilises a virtual environment and provides computer automated performance metrics and is considered an educational tool with great potential [2–7]. In recent years more realistic VR simulators have been developed for basic and advanced laparoscopic skills training.

The aim of this study was to determine the role of the CLSC in developing laparoscopic skills using the VR.

## 2. Design

**Figure 1:**
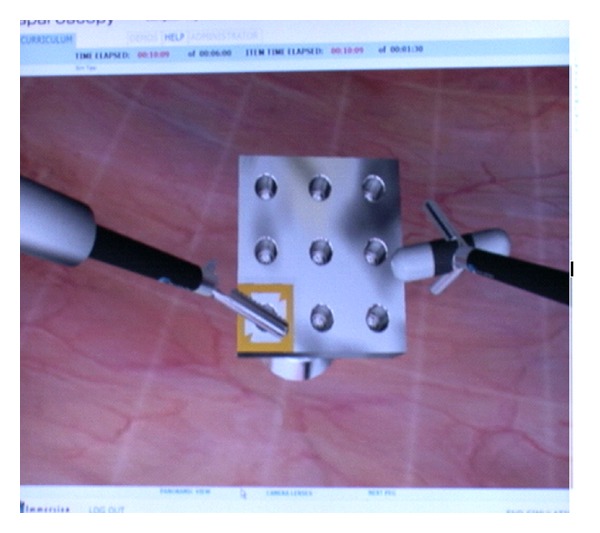
PEG transfer using the VR.

**Figure 2:**
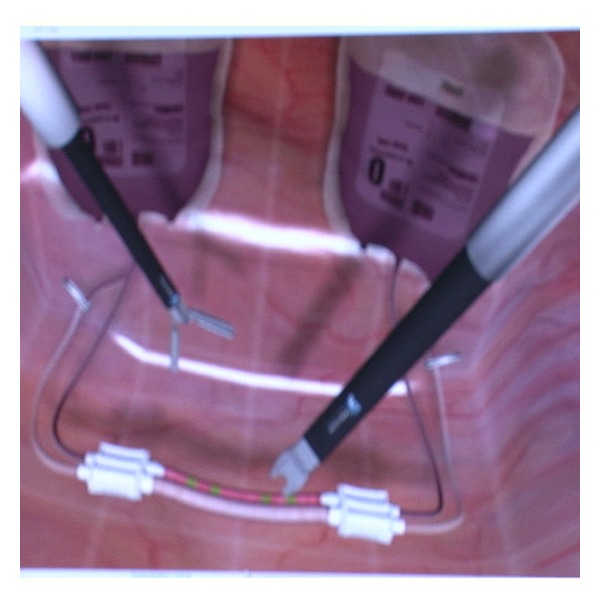
Clipping skills using the VR.

Between 2008 and 2010, doctors with minimal laparoscopic surgery experience attending the CLSC participated in this study. Initial teaching session (10–15 minutes) was given to explain how to perform PEG transfer (Figure 1) and clipping skills (Figure 2) using the VR.

The VR simulator used was *Immersion Virtual Laparoscopic Interface (Immersion Medical, Gaithersburg, MD)*. The PEG transfer requires the user to reach for and grasp a 3D simulated peg with an appropriate modelled instrument and place it into a predetermined hole in the simulated pegboard.

**Figure 3:**
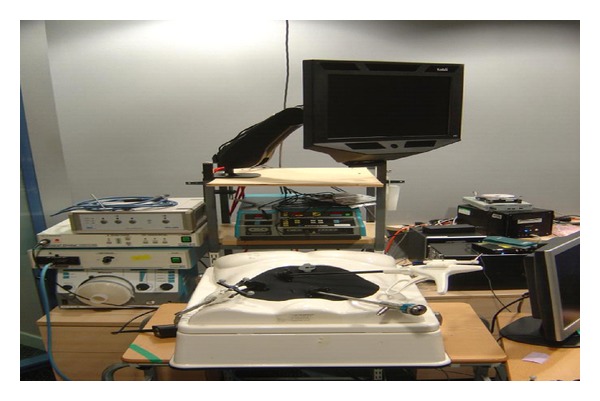
Traditional box [19, 20].

The candidates carried out these two skills before and after the course. The CLSC is an intensive practical skills course, accredited by the Royal College of Surgeons of England and taught by experienced laparoscopic surgeons. The course duration was 3 days and the candidates were trained using the BT (Figure 3) throughout the course. The course programme is shown in Table 1. The parameters assessed for PEG transfer included total time taken, total path length of right instrument, and total path length of left instrument. The parameters for clipping included total time taken, clips applied in marked area, cut within marked area, number of misplaced clips, and maximum vessel stretch.

## 3. Results

Forty-eight doctors were included in the study. Improvements were noted in various parameters after the core skills course as shown in Table 2.

**Figure 4:**
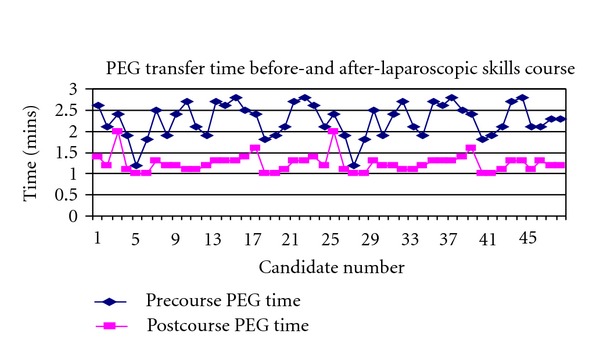
Time taken for PEG transfer.

**Figure 5:**
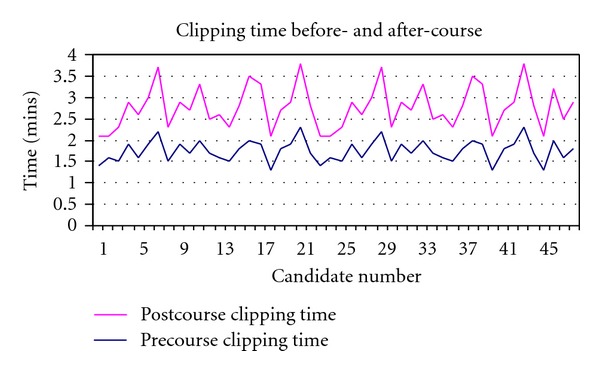
Time taken for clipping skills.

The mean time taken to complete the PEG transfer was 2.3 min before the course compared to 1.2 min after the course. The mean clipping skills time was 1.7 min and 1.0 min before and after the course. Figures 4 and 5 demonstrate the time taken for the PEG transfer and clipping skills.

**Figure 6:**
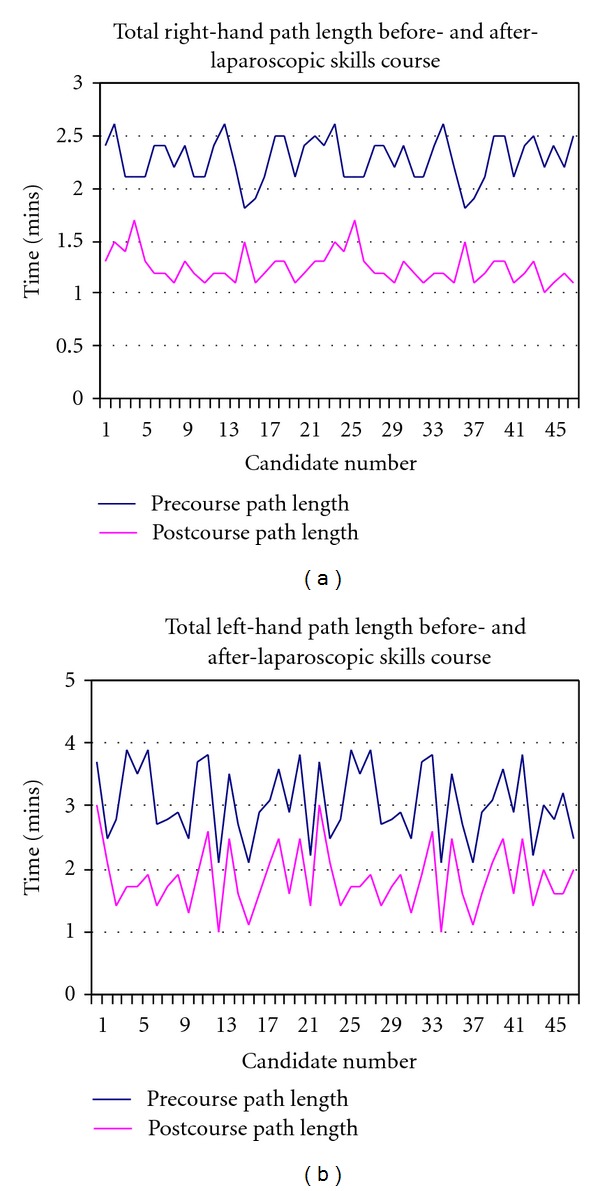
Total right- and left-hand length.

The mean total right- and left-hand path were 2.3 m and 3.0 m before the course and decreased to 1.3 m and 1.8 m, respectively, after the course (Figure 6).

## 4. Discussion

The CLSC is one of three laparoscopic courses accredited by the Royal College of Surgeons of England. It is ranked between the Basic Surgical Skills (BSS) course and the Advanced Laparoscopic Skills Course (ALSC). During the BSS course, the basic principals of laparoscopic surgery are taught on the last day of a 3-day course and the ALSC is mainly focused on suturing and anastomosis. We believe that the CLSC is suitable for the surgical trainees halfway through their training and it covers a large area of the Fundamentals of Laparoscopic Surgery Program [8].

Minimally invasive surgery has revolutionised surgical practise. Standard procedures like cholecystectomy and appendicectomy are often performed laparoscopically during the current practise. The current study showed that the CLSC improved certain laparoscopic skills like the PEG transfer and the clipping skills assessed by the VR.

With the advances in minimally invasive surgery [9, 10] as well as the European Working Time Directive leading to a reduction in working hours [11], there is an increased need for training out of the operating theatre. In our study, the time taken to complete the task has improved by nearly 50% over a short period of training (3 days) in the CLSC.

VR, with its many realistic properties, is not without their shortcomings. Many use the time taken to complete a task as the only objective measurement and fail to account for accuracy. This is common to training systems developed by Rosser et al. [12], SAGES [13], and Scott et al. [14]. Objective assessment of simulation performance is essential for laparoscopic skills acquisition. Without valid performance metrics, simulation training loses much of its credibility and value [15]. The VR we used in our study assessed the technical and dexterity skills as in the PEG transfer by measuring the total right- and left-hand length. It also measured the vessel stretch and the number of misplaced clips in the clipping skills.

Successful incorporation of simulator-based training in aviation [16] and limitation of the current student-mentor model [17] have led to emergence of surgical simulators. Limited studies assessed the validity of the VR [6]. Eriksen and Grantcharov [18] randomised 24 medical students to a practice-on-the-VR group or to a no-practice control group. They were evaluated performing tasks in a porcine model and the trained group did significantly better. In our study the candidates acted as their own control, they practiced on the BT during the CLSC whereas the evaluation was conducted by the VR. The results showed various aspects of laparoscopic skills improvement after the course.

Training laparoscopic courses have the potential to act as an adjunct to current training schemes in order to fully achieve surgical competence. They have been shown to develop surgical skill in a safe environment hence attending to current-day demands of training.

There was no control group for our study, as there were no candidates who underwent a pre- and post-course assessment, but did not actually undertake the course. This might be a limitation in our study.

This study demonstrated that CLSC improved some aspects of the laparoscopic surgical skills.

## Figures and Tables

**Table 1:** Course programme.

Day	Topic
1	Didactic lectures:
	History of laparoscopic surgery
	Physiological changes associated with pneumoperitoneum
	Simulator training:
	Instrumentation
	Safe access
	Manipulation
	Dissection

2	Didactic lectures:
	Appropriate use of energy source
	Complications, avoidance, and management
	Simulator training:
	Suturing and knotting
	Diagnostic laparoscopy
	Laparoscopic appendicectomy
	Repair of perforated duodenal ulcer

3	Simulator training:
	Laparoscopic cholecystectomy
	Continuous suturing

**Table 2:** Improved parameters after the course.

Skill	Improved by (%)
PEG:	
Total time to complete task	52
Total left hand length	41
Total right hand length	48
Clipping:	
Total time to complete task	57
Clips applied in marked area	38
Cut within marked area	42
Maximum vessel stretch	45
Number of misplaced clips	39
